# Effects of SGLT2 inhibitors on ion channels in heart failure: focus on the endothelium

**DOI:** 10.1007/s00395-025-01115-y

**Published:** 2025-05-14

**Authors:** Mengnan Wang, Benedikt Preckel, Coert J. Zuurbier, Nina C. Weber

**Affiliations:** Department of Anesthesiology – Laboratory of Experimental Intensive Care and Anesthesiology-L.E.I.C.A, Amsterdam University Medical Centers, Amsterdam Cardiovascular Science, Meibergdreef 9, 1105 AZ Amsterdam, The Netherlands

**Keywords:** Endothelial cells, Ion channels, Endothelial dysfunction, Vascular tone, Heart failure, Sodium-glucose cotransporter 2 inhibitors

## Abstract

Heart failure (HF) is a life-threatening cardiovascular disease associated with high mortality, diminished quality of life, and a significant economic burden on both patients and society. The pathogenesis of HF is closely related to the endothelium, where endothelial ion channels play an important role in regulating intracellular Ca^2+^ signals. These ion channels are essential to maintain vascular function, including endothelium-dependent vascular tone, inflammation response, and oxidative stress. Sodium-glucose cotransporter 2 inhibitors (SGLT2i) have shown promising cardiovascular benefits in HF patients, reducing mortality risk and hospitalization in several large clinical trials. Clinical and preclinical studies indicate that the cardioprotective effects of SGLT2i in HF are mediated by endothelial nitric oxide (NO) pathways, as well as by reducing inflammation and reactive oxygen species in cardiac endothelial cells. Additionally, SGLT2i may confer endothelial protection by lowering intracellular Ca^2+^ level through the inhibition of sodium-hydrogen exchanger 1 (NHE1) and sodium-calcium exchanger (NCX) in endothelial cells. In this review, we discuss present knowledge regarding the expression and role of Ca^2+^-related ion channels in endothelial cells in HF, focusing on the effects of SGLT2i on endothelial NHE1, NCX as well as on vascular tone.

## Introduction

Heart failure (HF) is a major cause of mortality and morbidity globally, with high prevalence, a poor prognosis, a negative impact on quality of life, and creates a heavy economic burden on both patients and the healthcare system [29, 66, 104, 168]. HF is differentiated based on left ventricular ejection fraction (LVEF): HF with reduced ejection fraction (HFrEF, LVEF ≤ 40%), HF with mildly reduced ejection fraction (HFmrEF, LVEF 41- 49%), and HF with preserved ejection fraction (HFpEF, LVEF ≥ 50%) [66, 104, 156]. Ischemic heart disease is the leading cause of HF, particularly HFrEF [75]. However, at least half of HF patients do not have a reduced LVEF [134]. The prevalence of HFpEF is increasing due to the rising prevalence of obesity and cardiometabolic disorders (e.g. type 2 diabetes mellitus (T2DM)) [134]. Approximately 84% of patients with HFpEF are readmitted to the hospital within 5 years, with nearly 40% of these readmission due to HF [143].

Right-ventricular HF resulting from right ventricular (RV) dysfunction received growing attention in recent years [85]. RV dysfunction can arise secondarily from left ventricular (LV) dysfunction, but also primarily by pathological processes affecting the RV and pulmonary vasculature (e.g. pulmonary hypertension) [60]. HF patients with RV dysfunction have worse outcomes compared to patients with preserved RV function [16]. Given these challenges, developing therapeutic drugs that can be useful in the broad spectrum of HF and improve ventricular function is of crucial importance.

Sodium-glucose cotransporter 2 inhibitors (SGLT2i) represent a class of oral anti-diabetic drugs mainly used for managing T2DM [71]. These drugs’ glycosuric function is mediated through inhibition of SGLT2, thereby blocking glucose reabsorption in the proximal tubular of kidneys [32, 139]. Although SGLT2i (e.g. empagliflozin (EMPA), canagliflozin (CANA), and dapagliflozin (DAPA)) were initially designed as glucose-lowering agents, they remarkably lower cardiovascular (CV) death and hospitalization in HF patients across the spectrum of LVEF, independent of their diabetic state [7, 106, 117, 125, 146, 171, 188]. Moreover, SGLT2i have been shown to improve RV function in HF patients, irrespective of the presence or absence of DM [1, 30]. However, their glycosuric effect cannot fully explain the cardioprotective effects of SGLT2i in patients with HF.

**Fig. 1:**
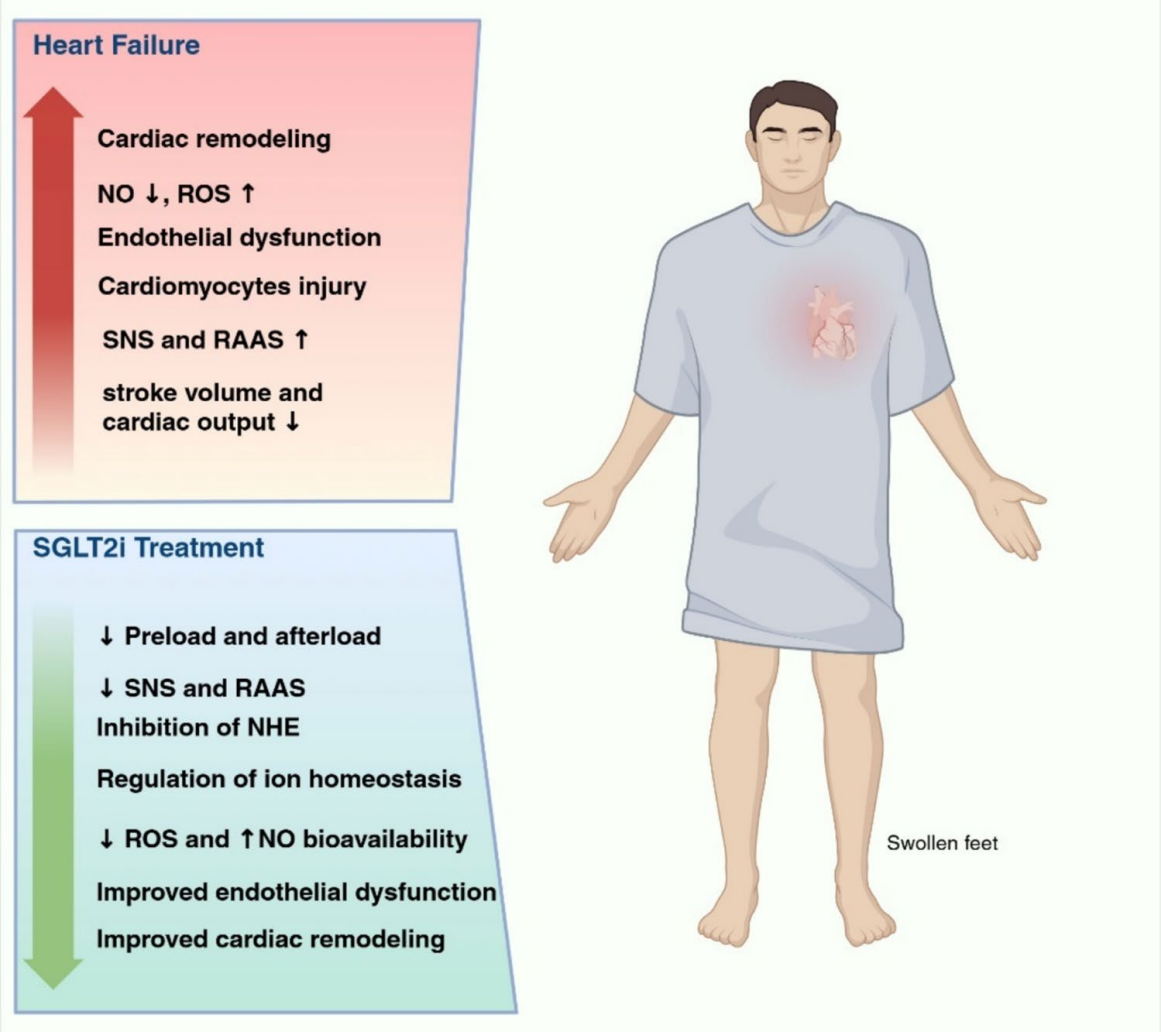
The mechanism of SGLT2i underlying HF. SGLT2i improve cardiac preload and afterload through their diuretic effect and inhibitory effect on SNS as well as RAAS. Furthermore, SGLT2i exert direct cardioprotective effects by enhancing NO bioavailability, reducing ROS generation and inhibiting cardiac NHE1 activity. *SGLT2i* sodium-glucose transporter inhibitors, *SNS* sympathetic nervous system, *RAAS* renin–angiotensin–aldosterone system, *NOS* nitric oxide synthase, *ROS* reactive oxygen species, *NHE* sodium-hydrogen exchanger

The clinical benefits of SGLT2i in HF are driven by mechanisms involving renal, vascular, cardiac, and systemic responses (thoroughly reviewed previously [131] and summarized in Fig.1). By inhibiting SGLT2 in proximal tubules, SGLT2i reduce sodium (Na^+^) reabsorption, promoting natriuresis and diuresis, which result in decreased plasma volume and subsequently improve cardiac preload. The natriuretic and diuretic effects of SGLT2i lower blood pressure, indicating the improvement of afterload. Beyond their diuretic effect, SGLT2i can also affect plasma volume and hemodynamics by modulating the sympathetic nervous system (SNS) and renin–angiotensin–aldosterone system (RAAS) activity [76].

The vascular endothelium covers the inner lining of blood vessels, and is vital in maintaining vascular homeostasis, including regulating blood flow, vascular permeability, vascular tone, inflammation and platelet activity [41, 89]. Due to the close proximity between cardiac endothelial cell (ECs) and neighboring cardiomyocytes (CMs), ECs release vasoactive factors (e.g. nitric oxide (NO)) to modulate CM contractility [2]. Under physiological conditions, NO diffuses into CMs, where it activates cGMP-protein kinase G (PKG), thereby regulating myocardial contractility and promoting vasodilation [49]. In HF, elevated inflammation and increased reactive oxygen species (ROS) production reduce NO bioavailability, leading to diminished PKG activity in CMs [127]. Low PKG activity impairs myocardial relaxation, resulting in compromised myocardial perfusion. Moreover, disruption of NO-cGMP-PKG pathway contributes to CM hypertrophy and the proliferation of myofibroblast, ultimately leading to LV dysfunction as well as fueling the progression of HF [49, 127]. The degree of endothelial dysfunction (ED) correlates with the severity of HF, and serves as an independent predictor of mortality in HF patients [41, 49]. ED is considered an early marker of HFpEF and has been observed in 29% of respective patients [49, 145]. Among the recommended medical therapies to address HFpEF, only SGLT2i have demonstrated a significant reduction in CV death in HFpEF patients [66, 105], implying that ED may be a key therapeutic target of SGLT2i in HF.

SGLT2i exert direct protective effects against HF-induced ED by enhancing NO bioavailability, promoting vasodilation, and reducing ROS generation (Fig. 1) [26, 44, 91, 158]. The beneficial effects of SGLT2i on in vivo HF were abolished under continuous NO inhibition by L-NAME, suggesting an important role of endothelial NO production in SGLT2i-mediated cardioprotection [26]. Moreover, the protective effects of SGLT2i against cardiac ischemia–reperfusion injury (IRI) are mostly related to EC protection rather than direct effects on CMs, indicating that ECs play a pivotal role in SGLT2i’s beneficial cardiovascular effects [119, 120]. These benefits may be initially mediated through endothelial ion channels that facilitate the maintenance of intracellular Ca^2+^ ([Ca^2+^]_i_) homeostasis. Our in vitro studies indicated that EMPA alleviated both inflammation- and mechanical force-induced ROS generation in human coronary artery ECs (HCAECs) by decreasing [Ca^2+^]_i_ through the inhibition of sodium-hydrogen exchanger 1 (NHE1) and sodium-calcium exchanger (NCX) activity [93–95, 160].

The present review focuses on the function of endothelial ion channels in the healthy and failing heart and summarizes findings from in vivo and in vitro studies on the effects of SGLT2i on endothelial ion homeostasis in HF.

## Endothelial function within the heart

The regulation of vascular tone is a central function of the endothelium. ECs produce and release vasoactive factors to constrict (e.g. endothelin-1) or relax (e.g. NO) blood vessels, thereby balancing oxygen supply and energetic metabolism [38]. In the healthy heart, ECs in coronary arteries and resistance vessels control coronary blood flow to meet the metabolic demand of the myocardium [67, 90]. ECs in the endocardium, due to their proximity to CMs, primarily regulate contractile performance and rhythmicity [21]. Many endothelial functions rely on [Ca^2+^]_i_, which is very low in resting ECs. In activated ECs, inflammatory mediators or mechanical forces activate phospholipase C (PLC) [37, 111], which subsequently cleaves phosphatidylinositol 4,5-bisphosphate (PIP2) into inositol-1,4,5-trisphosphate (InsP3) and diacylglycerol (DAG) [111]. InsP3 then triggers Ca^2+^ release from the endoplasmic reticulum (ER) through InsP3 receptors (InsP3R), leading to intracellular Ca^2+^ store depletion and Ca^2+^ entry from the extracellular space via transmembrane channels [37, 155]. To maintain resting [Ca^2+^]_i_ in ECs, elevated cytosolic Ca^2+^ quickly goes back to baseline via mitochondria and several ion channels, including sarco-endoplasmic reticulum Ca^2+^-ATPase (SERCA), plasma membrane Ca^2+^-ATPase as well as NCX [37, 110, 111].

**Fig. 2:**
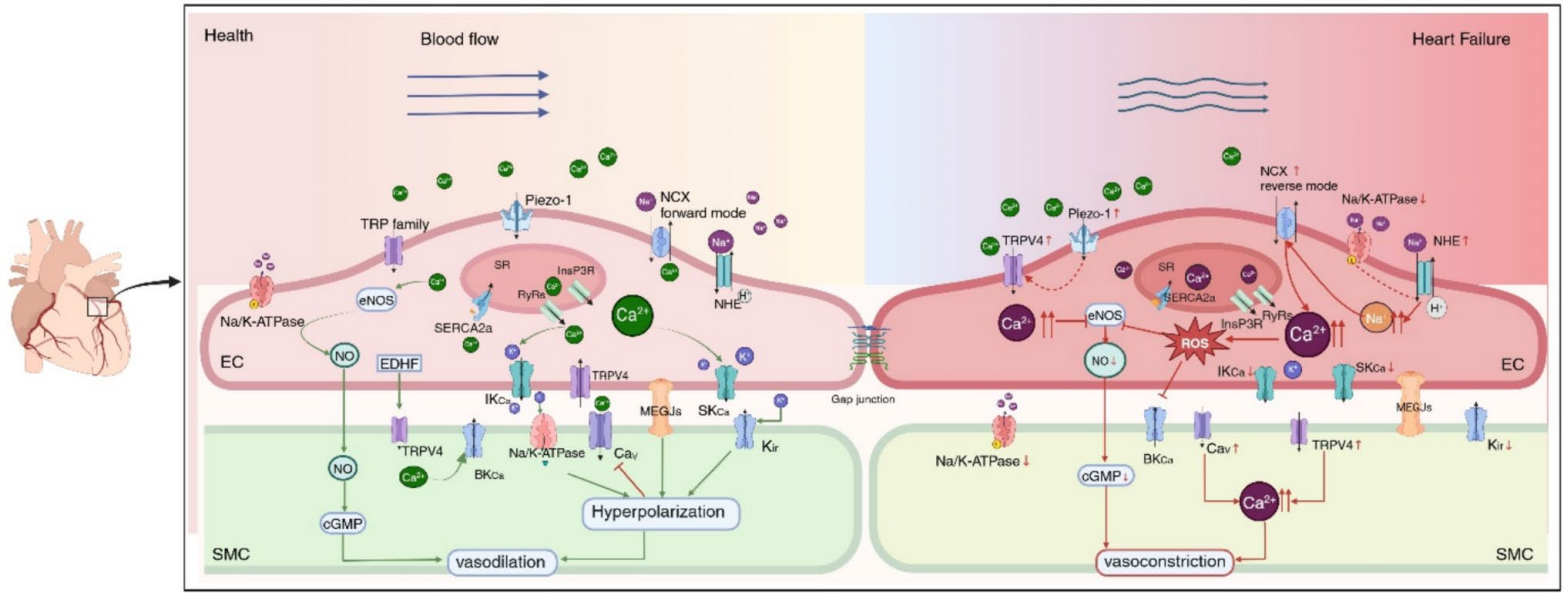
The role of endothelial ion channels in HF. ECs express many ion channels modulating ion homeostasis and are involved in several signaling transduction pathways. Healthy ECs secrete NO and EDHF to regulate vascular tone (left). Piezo-1 activates TRPV4 mediating the upregulation of [Ca^2+^]_i_. Increased [Ca^2+^]_i_ stimulates IK_Ca_ and SK_Ca_ in ECs to generate EDH which spreads to adjacent SMC via MEGJs, leading to vascular relaxation. In addition, NO diffuses to SMC activating cGMP, resulting in vasodilation. In the failing heart (right), changes in endothelial ion channels disrupt ion homeostasis. The activation of NHE increases [Na^+^]_i_ which triggers the reverse mode of NCX, leading to Ca^2+^ overload. Ca^2+^ overload inhibits IK_Ca_ and SK_Ca_ in ECs while promoting ROS generation. ROS overproduction deteriorates NO bioavailability, resulting in vasoconstriction. *EDH* endothelium-dependent hyperpolarization; [Ca^2+^]_i_: intracellular calcium concentration; *Ca*_*V*_ voltage-gated Ca^2+^ channels; *K*_*V*_ voltage-gated K^+^ channel; *NHE* Na^+^/H^+^ exchanger; *NCX* Na^+^/Ca^2+^ exchanger; *I*_*NaL*_ late sodium current; *MEGJs* myoendothelial gap junctions; *SK*_*Ca*_ small Ca^2+^-activated K^+^ channels; *IK*_*Ca*_ intermediate conductance Ca^2+^-activated K^+^ channels

Increased cytosolic Ca^2+^ activates calmodulin (CaM) binding to Ca^2+^/calmodulin-dependent kinase II (CaMKII), which subsequently increases endothelial nitric oxide synthase (eNOS) activity leading to NO production [6, 23, 184]. NO then diffuses into adjacent vascular smooth muscle cells (VSMCs) and binds to soluble guanylate cyclase (sGC). Activation of sGC stimulates cGMP production, which subsequently activates PKG, resulting in cytoplasmic Ca^2+^ decrease as well as smooth muscle relaxation (Fig.2) [38, 189]. NO regulates the expression of endothelial adhesion molecules and therefore, inhibits leukocyte recruitment and adhesion [77].

Next to NO, endothelium-dependent hyperpolarization (EDH) factors (EDHF) modulate endothelial-dependent relaxation in small resistance vessels when eNOS and prostacyclin are inhibited [54, 154, 163]. EDHF mediate vasodilation through hyperpolarization of VSMC. EDH is initiated by an elevation in endothelial [Ca^2+^]_i_ [56]. The increased [Ca^2+^]_i_ later activates small (SK_Ca_) and intermediate conductance (IK_Ca_) Ca^2+^ activated K^+^ channels in ECs [56]. These channels enable the transmission of hyperpolarization to smooth muscle cells (SMC) via myoendothelial gap junctions (MEGJs) [56]. In VSMC, K^+^ efflux activates Na^+^-K^+^-ATPase and inward-rectifier K^+^ channels, and closes voltage-sensitive Ca^2+^ channels (Ca_V_), leading to vascular relaxation [56, 84, 87]. The underlying mechanism described above is depicted in Fig. 2.

## The role of endothelial ion channels in HF

In HF, the release of inflammatory factors from myocardial tissue, coupled with altered shear forces, increases oxidative stress, and impairs endothelial function [122, 161]. Excessive ROS generation disrupts the function of endothelial ion channels, subsequently increasing [Ca^2+^]_i_ [118]. [Ca^2+^]_i_ overload stimulates downstream signaling pathways, causing the reduction of NO bioavailability, impaired endothelium-derived vascular relaxation, proinflammation and platelet aggregation [35, 113]. The decline in coronary endothelium-dependent vasodilation disrupts myocardial perfusion and exacerbates ventricular dysfunction [53].

The following section focuses on the role of endothelial ion channels in regulating vascular tone and Ca^2+^ homeostasis in the healthy heart and in HF (summarized in Fig. 2).

### TRPV4 channels

Transient receptor potential (TRP) channels are a group of non-selective cation channels classified into seven subfamilies: TRPC (canonical), TRPM (melastatin), TRPV (vanilloid), TRPP (polycystin), TRPA (ankyrin), TRPML (mucolipin), and TRPN (nompC) [14, 137]. Most of these channels are expressed in ECs and permeable to Ca^2+^, except for TRPM4 and -M5, which are permeable to Na^+^ and K^+^ [52, 149, 178]. These channels are differentially expressed in different organs, e.g. TRPA1 was expressed in cerebral arteries and was not detectable in mesenteric or coronary arteries [45, 150]. TRP channels function as cellular sensors, linking downstream Ca^2+^-sensitive signaling pathways and facilitating TRP-mediated Ca^2+^ influx to regulate endothelial function (Fig. 2) [109]. TRPP and TRPC channels are involved in endothelium-dependent vasodilation. A rise in extracellular Ca^2+^ concentration activated Ca^2+^ sensing receptors, inducing Ca^2+^ influx through TRPC1 [58, 70, 132]. TRPC1-mediated Ca^2+^ influx led to NO production in the endothelium and subsequent vasorelaxation. Endothelial cell-specific TRPC1 knock-out (KO) mice had lower EF and larger myocardial infarct size after left anterior descending (LAD) coronary artery ligation-induced myocardial infarction (MI) [169]. Enhanced TRPC1 expression alleviated MI-induced ischemia by increasing capillary density [169]. Moreover, TRPP2 formed a complex with TRPC1 and TRPV4 in cultured rat mesenteric artery ECs, as a response to mechanical forces [151]. Transfection of rat mesenteric artery ECs with nonfunctional pore mutant of either TRPP2, TRPC1 or TRPV4 abolished shear stress-induced Ca^2+^ influx and subsequent vascular dilation, suggesting that the TRPV4-TRPC1-TRPP2 complex mediated flow-induced Ca^2+^ influx and vasorelaxation in vascular ECs [42]. Among TRP channels, endothelial TRPV4 has received increasing attention in cardiovascular diseases due to its wide expression in cardiac ECs, high Ca^2+^ permeability, and strong interaction with other ion channels [55].

TRPV4 is essential to vascular tone. Lu et al. exposed human umbilical vein ECs (HUVECs) to sodium chloride (NaCl, 5 mM) to investigate the mechanism underlying Cl^−^-induced endothelium-dependent vasorelaxation [99]. Cl^−^-induced vasodilation mainly occurs through EDH, which is initiated by increased [Ca^2+^]_i_ in ECs [99]. In HUVECs, Cl^−^-mediated TRPV4 activation induced increased [Ca^2+^]_i_, leading to vasodilation. In contrast, mesenteric arteries isolated from TRPV4 KO mice lost the ability to respond to Cl^−^mediated vasodilation, suggesting that TRPV4-mediated intracellular Ca^2+^ elevation stimulated vasodilation [99]. This finding is consistent with data showing that resting blood pressure was significantly higher in endothelial-specific TRPV4 KO mice than in wild-type (WT) mice [123]. The activation of endothelial TRPV4 triggered downstream SK_Ca_ and IK_Ca_, leading to EDH-mediated vasodilation [141]. Isometric tension recording and electrophysiological experiments on SMC membrane potential were used to distinguish whether acetylcholine (Ach, an activator of eNOS)-mediated vasodilation was dependent on NO or hyperpolarization. Blocking TRPV4 abolished Ach-induced EDH-mediated vasodilation in mesenteric arteries but did not affect NO-dependent relaxation. TRPV4 activation increased hyperpolarization, which was attenuated by inhibiting SK_Ca_ or IK_Ca_, with further reduction observed when both SK_Ca_ and IK_Ca_ were inhibited [141]. These data indicated that TRPV4-induced EDH was mediated by increasing intracellular Ca^2+^, which was initiated downstream of SK_Ca_ and IK_Ca_. Since TRPV4 is expressed in both ECs and SMC, the investigators removed the endothelium showing that endothelial denudation eliminated TRPV4 activation-mediated vasodilation. Furthermore, the expression of endothelial TRPV4 was reduced in mesenteric arteries from hypertensive rats. Taken together, these data suggested that the endothelial TRPV4, but not SMC TRPV4, contributed predominantly to vasodilation [141]. These results were confirmed in human coronary arteries (HCA) where arachidonic acid caused HCA dilation [185]. Arachidonic acid-induced relaxation was suppressed in the presence of TRPV4 antagonist as well as the inhibitors of IK_Ca_ and SK_Ca_ channels. Moreover, an in vitro study elicited that the forward mode of NCX extruded TRPV4-induced elevated intracellular Ca^2+^ in ECs, indicating that endothelial Ca^2+^ homeostasis was maintained by TRPV4/NCX [99].

The function of endothelial TRPV4 in hypertension is bidirectional [182]. Hypertension increased endothelial TRPV4 expression compared to control rats, while SMC showed a low level of TRPV4 expression. Activating TRPV4 with low concentrations of TRPV4 activator GSK1016790A (GSK, 1–10 nM) in mesenteric arteries from hypertensive rats exerted vasorelaxation, but high doses (20–30 nM) of GSK exerted vasoconstriction. Notably, GSK did not induce constriction in endothelium-removed arteries nor did it increase Ca^2+^ in isolated SMC, suggesting that TRPV4 is involved in endothelium-dependent regulation of vascular tone. GSK elevated intracellular Ca^2+^ in ECs from hypertensive rats in a concentration-dependent manner. One potential explanation for TRPV4-induced vasocontraction was that overactivation of TRPV4 evoked sustained Ca^2+^ and excessive mitochondrial Ca^2+^ uptake, resulting in the upregulation of ROS generation [182]. ROS overproduction thereby disrupted NO formation and promoted the release of endothelium-derived contracting factors.

In addition to regulating vascular tone, the endothelial TRPV4 channel is a critical regulator of cardiac fibrosis in HF [107, 133]. Adapala et al. established an endothelial-specific deletion of TRPV4 mice (TRPV4^ECKO^, TRPV4^lox/lox^ as comparison) to investigate the function of endothelial TRPV4 channels in transverse aortic constriction (TAC)-induced HF [3]. Compared to TRPV4^lox/lox^ mice, TRPV4^ECKO^ mice exhibited attenuated cardiac fibrosis and cardiomyocyte apoptosis following TAC. The coronary capillary density was increased in TRPV4^ECKO^ after TAC. These results illustrated that endothelial TRPV4 deletion attenuated TAC-induced cardiac fibrosis and cardiomyocyte apoptosis, and enhanced coronary angiogenesis [3]. An increased endothelial TRPV4 expression was observed in lung sections from HF patients compared to those without HF [153]. To further explore the function of endothelial TRPV4 in HF-induced pulmonary edema, HUVECs as well as isolated mouse lungs were treated with TRPV4 activator, resulting in pulmonary edema and reduced endothelial monolayer resistance (barrier integrity). These deteriorating effects were abolished by TRPV4 blockade, suggesting that HF-induced TRPV4 activation compromised endothelial barrier function, eventually leading to lung edema [153]. Recently, a selective TRPV4 blocker GSK2798745 revealed beneficial effects on lung gas transfer in phase II_a_ clinical trials in patients with HF [57, 148].

Taken together, the endothelial TRPV4 channel is a potential target in HF by reducing cardiac dysfunction and regulating vascular tone (Fig. 2). Furture studies should elucidate how endothelial TRPV4 regulate vascular tone under pathophysiological conditions.

### Piezo-1 channel

Piezo-1 is a mechanosensitive cation channel mediating cation current and Ca^2+^ signaling in various cell types [78]. Piezo-1 is expressed in cardiovascular cells including cardiac ECs, CMs, and fibroblasts [15, 31, 180]. As a cellular mechanosensor, Piezo-1 converts mechanical impulses to electrical or chemical signals and plays a critical role in cardiovascular physiology and pathophysiology [31, 180].

Hypertension is a leading cause of HF [40]. Adequate blood pressure management can reduce the risk of HF [47, 50]. Endothelial Piezo-1 is essential for the regulation of vascular tone and blood pressure [79]. Laminar shear stress activates endothelial Piezo-1 channels driving Ca^2+^ entry and stimulating eNOS phosphorylation (at serine 1177), which leads to upregulation of NO, resulting in vascular relaxation [15, 92]. The underlying mechanism of endothelial Piezo-1-dependent vasorelaxation might involve flow-induced adenosine triphosphate (ATP) release and subsequent activation of G proteins Gq/G_11_-coupled purinergic P2Y_2_ receptor by endothelial Piezo-1 [164]. The activation of P2Y_2_/Gq/G_11_ phosphorylates AKT and eNOS, resulting in NO formation as well as vasodilation [164, 165]. Mice with endothelium-specific Piezo-1 deficiency lost the ability to produce NO and consequently developed hypertension [164].

Piezo-1 senses also disturbed blood flow which induced vascular pathological responses leading to atherosclerosis [79]. Disturbed flow activated inflammatory signaling pathways in vascular ECs that increase the expression of leukocyte adhesion molecules and impair endothelial function [9]. ED is seen as an early step in the development of atherosclerosis [128]. In atherosclerotic mice, Piezo-1 was overexpressed and mainly colocalized with ECs in atherosclerotic lesions [88]. Inhibition of Piezo-1 by a specific inhibitor or endothelium-specific Piezo-1 knockdown significantly reduced the progression of carotid plaque lesions and suppressed the expression of atherosclerotic inflammatory markers, vascular cell adhesion protein-1 (VCAM-1) and intercellular adhesion molecule-1 (ICAM-1) [4, 88]. In vitro, oscillatory shear stress promoted intracellular Ca^2+^ accumulation through Piezo-1 activation, which potentially initiated downstream focal adhesion kinase, leading to the activation of NF-κB and yes-associated protein, thereby inducing endothelial inflammation [4, 88]. Endothelial Piezo-1 knockdown strongly reduced disturbed flow-mediated inflammatory response [4, 88], indicating the key role of Piezo-1 in endothelial inflammation.

Taken together, endothelial Piezo-1 plays an important role in maintaining basal blood pressure and mediates anti-inflammatory effects in response to laminar shear stress. However, the role of endothelial Piezo-1 in the pathology of HF is still unclear.

### Sodium-calcium exchanger (NCX)

Sodium-calcium exchanger (NCX) is widely expressed in cardiac ECs and plays a pivotal role in intracellular Ca^2+^ homeostasis of ECs [17, 83]. NCX is a bidirectional electrogenic transporter. Normally, NCX extrudes one Ca^2+^ from the cell, with three Na^+^ entering the cell, called the forward mode. When membrane depolarization is coupled with high [Na^+^]_i_, elevated [Na^+^]_i_ triggers the reverse mode of NCX (NCXrm), leading to Ca^2+^ influx and Na^+^ extrusion from the cell [18, 144]. NCX is considered to have an important role in endothelium-dependent vasodilation by regulating NO production [140].

Inhibition of NCXrm abolished Ach-mediated endothelium-dependent vasodilation and NO production in mesenteric arteries, while having no effect on endothelium-independent vasodilation [97]. Immunofluorescence staining further revealed that NCX colocalized with eNOS in caveolae [97], which are flask-shaped invaginations of the plasma membrane that regulate vascular tone by activating eNOS in ECs [28]. These findings suggest that NCXrm increased [Ca^2+^]_i_, thereby triggering eNOS/NO, leading to endothelium-dependent vasodilation. In TAC-induced cardiac hypertrophic rats, the expression of NCX and eNOS was reduced in the aortic endothelium, whereas NCX expression in the myocardium was slightly elevated but not significantly different from that in sham rats [10]. Treatment with curcumin, an active component in turmeric with anti-inflammatory and antioxidant effects, increased the expression of NCX and eNOS in both the aortic endothelium and myocardium of TAC-induced rats [10]. Curcumin treatment also improved vasorelaxation as well as cardiac function compared to untreated TAC rats [10]. Furthermore, pretreatment of aortic endothelium with KB-R7943, a selective NCXrm inhibitor, partially blocked the beneficial effect of curcumin on vasodilation, further suggesting the central role of NCXrm in endothelium-dependent vasodilation [10]. Although this study indicated that increased NCX expression probably improved TAC-induced cardiac function and vascular function, the authors did not investigate the mode of NCX (forward or reverse), or the location of NCX within the myocardial samples (cardiac ECs and/or CMs). Moreover, while NCXrm expression was associated with improved vascular function, its direct role in cardioprotection remains unclear. In contrast, upregulated NCX activity has been recognized as a mechanism promoting HF [130].

High glucose decreased Ach-mediated endothelium-dependent relaxation in the aorta rings from diabetic rats, compared to aorta rings from non-diabetic rats [96]. Superoxide dismutase (SOD, an enzymatic antioxidant) activity and NO level were reduced in the plasma of diabetic rats as well as in high glucose-induced HUVECs [96, 98]. In addition, high glucose upregulated NCX activity in HUVECs [98]. Treatment with KB-R7943 decreased NCX activity, increased SOD activity and NO release, and markedly enhanced Ach-mediated relaxation in diabetic aorta rings [96, 98]. These results suggested that inhibition of NCXrm restored impaired endothelium-dependent vasodilation induced by diabetes, which might be through scavenging ROS and enhancing NO production [96]. Moreover, a recent study from our group further confirmed the central role of increased NCX activity in oxidative stress. ROS generation was strongly increased in HCAECs exposed to 10% cyclic stretch (a pathological condition), while inhibiting NCX by either NCX knockdown or a specific NCX inhibitor, ORM-10962, abolished ROS production [94].

Future studies need to focus on the mode shift of NCX in HF to elucidate its role in HF-induced ED.

### Sodium-hydrogen exchanger (NHE)

The sodium-hydrogen exchanger (NHE) maintains intracellular pH and [Na^+^]_i_ by exchanging one intracellular H^+^ for one extracellular Na^+^ [138]. Ten isoforms of NHE have been identified, from which NHE1 has been widely studied [137, 175] and is the primary isoform in the heart [124]. In HF, NHE1 activation increases [Na^+^]_i_ which stimulates the reverse mode of NCX, leading to Ca^2+^ overload and causing ED (Fig. 2) [116, 175].

NHE1 activation is associated with damaged vascular tone. Rabbits fed either a normal or an atherogenic diet were used to assess vascular function, showing that endothelium-related vasodilation in response to Ach was blocked in thoracic aorta rings of hypercholesterolemic rabbits [80]. Treatment with the NHE1 inhibitor cariporide for 4 weeks strongly prevented impaired endothelium-dependent relaxation induced by hypercholesterolemia [80]. The beneficial effects of NHE1 inhibition on endothelium-dependent vasorelaxation were further confirmed in the study of coronary arteries [162]. Hearts were collected from either control, streptozotocin-induced diabetes, or diabetes treated with cariporide rats, and rapidly perfused according to Langendorff mode. Endothelium-dependent dilation in coronary arteries was estimated by injecting carbamoylcholin (an Ach analogue), which acts directly on ECs and produces NO [162]. Compared to the control group, diabetes significantly impaired endothelium-dependent dilation in coronary arteries. The inhibition of NHE1 by cariporide significantly alleviated impaired vasorelaxation induced by diabetes [162]. In addition, overactivated NHE1 by NHE activator LiCl inhibited endothelium-dependent vasodilation in rat aorta rings, but did not affect endothelium-independent vasodilation [73, 187]. These data suggested that the protective effect of NHE inhibition on vascular tone was mainly dependent on endothelium, probably through the modulation of the eNOS/NO pathway [73, 166, 187].

NHE1 is involved in the regulation of oxidative stress and inflammation. In human cardiac microvascular ECs stimulated with uremic serum from patients with chronic kidney disease (CKD), treatment with cariporide inhibited ROS generation induced by uremic serum [81]. A potential mechanism underlying NHE-mediated ROS generation was identified in a dynamic HCAEC model, where 10% cyclic stretch induced ROS overproduction, probably through activation of the NHE1/Ca^2+^/NCX/PKC/NOX axis [94]. Increased NHE1 activity induced by cyclic stretch triggered downstream NCX activation, leading to intracellular Ca^2+^ accumulation. The upregulation of [Ca^2+^]_i_ activated the PKC/NOX (protein kinase C/ nicotinamide adenine dinucleotide phosphate oxidase) axis resulting in ROS generation [94]. In addition, NHE1 blockade attenuated the inflammatory response by inhibiting NF-κB activation, reducing ICAM-1 expression and preventing endothelial glycocalyx degradation in ECs [65, 173, 181]. HUVECs were exposed to low shear stress (LSS, < 5 dyn/cm^2^) to mimic early atherosclerosis-induced ED [181]. LSS elevated NHE1 activity, induced endothelial glycocalyx degradation, and suppressed AMP-activated protein kinase (AMPK) activity. AMPK activation prevented LSS-induced glycocalyx impairment and decreased NHE1 activity, while inhibiting NHE1 did not reverse LSS-induced AMPK inactivation. This suggested that AMPK regulates the glycocalyx via the modulation of downstream NHE1 activity [181].

Given the important role of NHE1 in vascular tone, inflammatory response as well as oxidative stress in ECs, more studies are required to further investigate the involvement of NHE1 in ED in HF.

### Ca^2+^-activated K^+^ channels (K_Ca_)

Ca^2+^-activated K^+^ channels (K_Ca_) are classified into three subtypes including the large conductance K_Ca_ channels (BK_Ca_), SK_Ca,_ and IK_Ca_ [5]. BK_Ca_ channels are predominantly expressed in VSMC [59, 129]. In VSMC, EDHF activates TRPV4 with increasing Ca^2+^ influx and then stimulating SR generating Ca^2+^ spark, which in turn activates neighboring BK_Ca_ resulting in hyperpolarization and blunting of the inherent pressure-induced constriction (Fig. 2) [24]. The expression of BK_Ca_ in ECs is controversial. These channels were repeatedly reported as expressed in cultured ECs [136]. In cultured bovine aortic ECs, BK_Ca_ channels were localized to caveolae microdomains and were negatively regulated by caveolin [167]. Electrophysiological studies in the freshly isolated bovine coronary artery, mouse carotid artery, as well as rat cerebral parenchymal arteriolar cell only revealed SK_Ca_ and IK_Ca_ current, but no BK_Ca_ current was measured [20, 51, 63, 74, 167]. This indicates that the expression of BK_Ca_ channels in ECs probably depends on specific conditions or tissues being studied [20, 51, 63, 74, 167].

Activation of SK_Ca_ and IK_Ca_ leads to hyperpolarization of ECs which subsequently hyperpolarizes the adjacent VSMC, resulting in vasodilation (Fig. 2) [33, 176]. Changes in the expression and function of SK_Ca_ and IK_Ca_ have been reported in cardiovascular disease. Hypertensive rats showed an impaired EDH-mediated response in mesenteric arteries as a result of the reduction in endothelial SK_Ca_ expression, suggesting a relevance of SK_Ca_ expression in hypertension [69, 141]. In human atrial tissue subjected to cold ischemia/reperfusion, the function of endothelial SK_Ca_ and IK_Ca_ channels was impaired, resulting in microvascular dysfunction [48]. Yet preserved and augmented function and expression of IK_Ca_ channels have been observed in hypertensive animals [59, 68, 112], suggesting that the function of SK_Ca_ and IK_Ca_ might be bidirectional. Given the conflicting findings regarding the functions of SK_Ca_ and IK_Ca_ channels in ECs, additional research is needed to elucidate the mechanisms that drive these changes in K_Ca_ channels.

## The effect of SGLT2i on endothelial ion channels

**Fig. 3:**
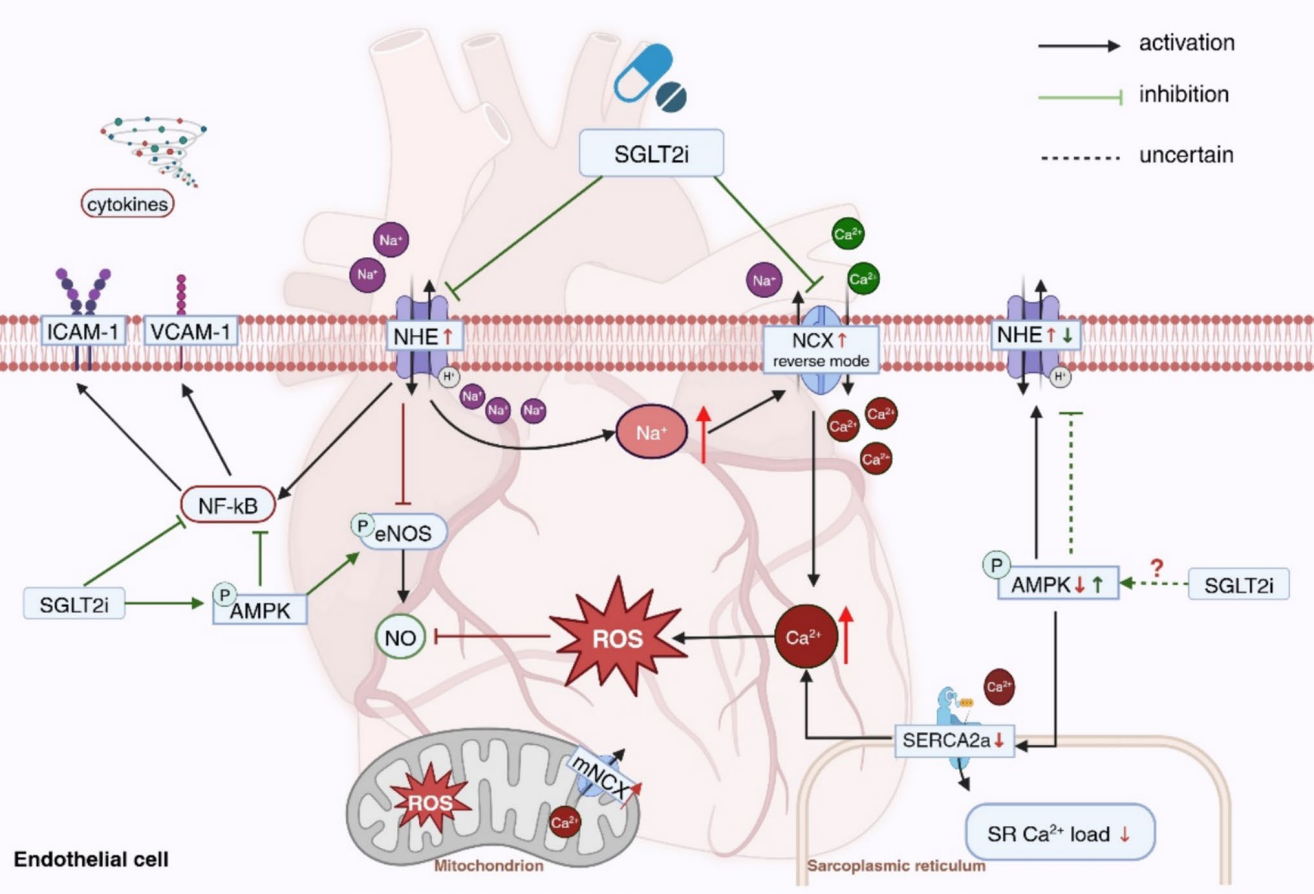
The effects of SGLT2i on endothelial ion channels. SGLT2i decrease intracellular Na^+^ and Ca^2+^ overload by directly or indirectly inhibiting NHE1 and NCX. A reduction in Ca^2+^ inhibits ROS generation. In addition, SGLT2i alleviate endothelial inflammation as well as impaired eNOS activity, which might be through the NHE1 inhibition and AMPK activation. *NHE* Na^+^/H^+^ exchanger; *NCX* Na^+^/Ca^2+^ exchanger; *mNCX* mitochondria Na^+^/Ca^2+^ exchanger; *ROS* reactive oxygen species; *AMPK* AMP-activated protein kinase

Since SGLT2i have been reported to improve cardiovascular prognosis, many preclinical studies investigated the mechanisms underlying the clinical benefit of SGLT2i in HF. The imbalance of ion homeostasis contributes to cardiac dysfunction in HF, and SGLT2i have been reported to regulate cytosolic Na^+^ and Ca^2+^ (the potential mechanisms are depicted in Fig.3).

### The effect of SGLT2i on NHE

As discussed in Sect. "Sodium-hydrogen exchanger (NHE)", overactivation of endothelial NHE1 plays a key role in inflammatory response, ROS production, and the impairment of vascular tone induced by HF. Some of the cardioprotective effects of SGLT2i in HF are probably mediated by inhibiting cardiac NHE1 [8].

Isolated mouse hearts were perfused with SGLT2i for 30 min, revealing that EMPA (1 µM) and CANA (3 µM), but not DAPA (1 µM), induced coronary vasodilation in the healthy mouse heart [157]. The molecular docking model found that SGLT2i (EMPA, CANA, and DAPA) had a high binding affinity to the extracellular Na^+^-binding site of NHE [157], suggesting that the beneficial effect of SGLT2i on coronary vasodilation might be achieved through the inhibition of NHE1 in ECs. Although short-term DAPA treatment did not affect coronary vasodilation, long-term treatment with DAPA seemed to regulate eNOS expression in the myocardium. In salt-sensitive rats fed a high-salt (HS) diet to mimic hypertensive-induced HFpEF, the HS diet caused diastolic dysfunction, cardiac inflammation, and a reduction in eNOS expression in the myocardium [22]. Immunofluorescence staining revealed a marked increase in both NHE1 and phosphorylated NF-κB expression in the coronary endothelium of HS rat hearts. Treatment with DAPA (0.1 mg/kg/day) for 6 weeks alleviated HS-induced diastolic dysfunction, reduced inflammation and NHE1 expression, and restored eNOS levels in the myocardium [22]. These findings implied that the cardioprotective effects of DAPA in HS-induced HFpEF may be mediated via inhibiting NHE1 and increasing eNOS in cardiac ECs.

An increase in NO level can not only occur due to the production of NO but also by a reduction in NO scavenging. TNF-α increased ROS generation in ECs by elevating [Na^+^]_i_ via the activation of NHE1 [160]. Treatment with EMPA (1 µM) significantly reduced TNF-α-induced increases in [Na^+^]_i_, NHE1 activity, as well as ROS production. Furthermore, the combination of EMPA with the NHE1 inhibitor cariporide did not further lower ROS production, indicating that EMPA inhibited NHE1/Na^+^, leading to decreased ROS generation [160]. In co-cultures of CMs and coronary microvascular ECs (CMECs) exposed to uremic serum from patients with CKD, uremic serum impaired CMEC-mediated CM relaxation, reduced endothelial NO levels, and increased ROS production [81]. EMPA (1 µM) enhanced CMEC-mediated CM dilation and endothelial NO levels, and reduced the level of ROS. However, neither the expression of total eNOS nor the phosphorylation of eNOS (Ser1177) was altered by EMPA, suggesting that EMPA preserved endothelium-dependent vasodilation by inhibiting ROS and, therefore, mitigating the scavenging of NO [81]. The NHE1 inhibitor cariporide (10 µM) lowered uremic serum-mediated ROS production. Combining EMPA with cariporide reduced ROS levels more extensively (EMPA and cariporide 75% vs cariporide 38%) in CMECs exposed to uremic serum. These data indicated that the protective effect of EMPA on endothelium-dependent vasodilation was primarily mediated through mitigating ROS overproduction, with this mechanism being at least in part independent of NHE inhibition. Supporting this, in HCAECs subjected to cyclic stretch (10% stretch as a pathophysiological condition), co-treatment with EMPA (1 µM) and cariporide (10 µM) exhibited a greater reduction in Ca^2+^ influx than cariporide alone [94]. It suggested that in addition to NHE1, the endothelial protective effect of EMPA may also be mediated by other ion channels.

Taken together, these findings indicate that the endothelial protective effect of EMPA may be, at least in part, dependent on NHE1. Further studies are required to elucidate the correlation of SGLT2i with NHE1-mediated endothelial dysfunction.

### The effect of SGLT2i on NCX

As discussed above, co-treatment with EMPA and cariporide reduced Ca^2+^ influx more effectively than cariporide alone in HCAECs [94]. To investigate the potential ion channel involved in EMPA-mediated [Ca^2+^]_i_ downregulation, the NCX was inactivated in HCAECs, given that the reverse mode of NCX is usually triggered by NHE1-induced [Na^+^]_i_ accumulation. The combination of EMPA and NCX inhibitor ORM-10962 did not cause further reduction in Ca^2+^ when compared to EMPA alone, suggesting that EMPA lowered Ca^2+^ in HCAECs via inhibition of NCX [94]. In order to further verify the interaction between NHE1, [Na^+^]_i,_ and NCX in endothelial [Ca^2+^]_i_, intracellular Ca^2+^ concentration was measured in NCX knockdown HCAECs in the presence of cariporide or Ouabain (a sodium/potassium pump inhibitor). Ouabain strongly upregulated [Ca^2+^]_i_ in wild-type HCAECs, and the elevated [Ca^2+^]_i_ was abrogated by NCX knockdown. In addition, cariporide slightly reduced intracellular Ca^2+^ in wild-type HCAECs, while NCX knockdown exerted a further reduction in [Ca^2+^]_i_ in HCAECs receiving cariporide (WT + cariporide vs. NCX KD + cariporide). The inhibition of NHE1 did not reduce the intracellular Ca^2+^ in NCX knockdown ECs. Taken together, these data suggested that NHE1 activation-induced [Na^+^]_i_ overload triggered downstream NCX, which subsequently increased [Ca^2+^]_i_, thereby leading to ROS overproduction. Therefore, the inhibitory effect of EMPA on ROS generation is probably mediated by inhibiting NHE1/Na^+^/NCX/Ca^2+^ axis [94].

### The effect of SGLT2i on vascular tone

The clinical effect of SGLT2i on regulation of blood pressure has been widely reported [61], however, the extent to which their antihypertensive effects contribute to SGLT2i-improved clinical outcomes in HF remains uncertain. On average, SGLT2i reduce systolic and diastolic blood pressure by approximately 4 and 2 mmHg, respectively [13, 82]. Böhm et al. demonstrated that SGLT2i reduced systolic blood pressure slightly more in HFpEF patients with resistant hypertension than in none-resistant hypertensive HFpEF patients, whereas the SGLT2i treatment effect was similar between the different categories of hypertensive HFpEF patients, indicating that the moderate modifications of blood pressure did not affect the therapeutic effects of EMPA in HFpEF [19].

In addition to NO-dependent vasodilation, endothelial potassium (K^+^) channels play an important role in the modulation of blood pressure and coronary blood flow. K^+^ released from ECs contributes to the endothelium-dependent relaxation, being a component of endothelium-derived hyperpolarization factor-mediated responses [62]. Activation of endothelial K_Ca_ channels has been shown to mitigate coronary endothelial dysfunction and preserve coronary flow in diabetic myocardium [108, 183].

In vivo studies have been implemented to elucidate the relation between SGLT2i and K^+^ channels. CANA caused mesenteric artery vasodilation at 1 μM as well as 10 μM, which was in line with the therapeutic concentration in patients (EC_50_ of CANA in patients is 11.21 μM) [64]. To clarify the involvement of endothelium in CANA-mediated vasodilation, mesenteric arteries were exposed to either an NO inhibitor, eNOS inhibitor, or prostacyclin (another endothelium-derived vasodilator) inhibitor, showing that none of these inhibitors affected CANA-mediated vasorelaxation. CANA-induced vasodilation still existed after the removal of the endothelium, suggesting that CANA-influenced vasodilation was independent of endothelium, probably through the regulation of K^+^ channels in SMC [64]. In addition, the effect of EMPA on vascular tone were assessed in patients with T2DM receiving either EMPA (10 mg per day) or a placebo [86]. Vascular function was measured by venous occlusion forearm plethysmography (VOP), an invasive method that can distinguish endothelium-dependent and endothelium-independent vasodilation via intra-arterial infusion of vasoactive drugs (Ach and SNP, NO donor) [86]. Fifteen participants were included for vasodilation analysis. Compared to the placebo group, EMPA treatment for 4 weeks improved vascular increased SNP response by 21%, while Ach response rose by 9% [86]. Plasma nitrite and cGMP were measured to further test endothelium-dependent vasodilation. EMPA did not change nitrite and cGMP levels in plasma compared to placebo, but even slightly reduced the plasma nitrite level [86]. These clinical findings align with the study by Hasan et al. showing that SGLT2i-induced vasorelaxation may directly act on SMC rather than ECs [64, 86]. In contrast, Sposito et al. showed that DAPA increased plasma nitrite level during flow-mediated dilation (FMD) measurement ( a non-invasive method for vascular function) in patients with T2DM, indicating the vasorelaxation effect of DAPA being dependent on the endothelium [147]. Similarly, EMPA (25 mg per day) intervention following STEMI (ST-segment elevation myocardial infarction) improved FMD of the brachial artery in T2DM patients [120]. The difference between these studies may be explained by the change in blood flow. FMD measurement is widely regarded as a method that reflects endothelium-dependent vascular function [152]. During FMD, an increase in blood blow and shear stress can activate ECs to produce NO. In contrast, blood collected before VOP is in a relatively steady state [86]. Although improvement of FMD in the forearm following SGLT2i treatment provides stronger evidence for enhanced endothelial function, there is still no direct proof of better coronary endothelial function [68]. There is a lack of research regarding whether SGLT2i affect the activity or expression of these endothelial K^+^ channels. Therefore, in vivo as well as in vitro (co-cultured coronary artery ECs and CM) studies are required to elucidate the mechanisms by which SGLT2i regulate vascular tone.

## Future perspectives

Endothelial dysfunction occurs not only following HF but also before the development of HF as an independent predictor of the incidence of HF [102]. Under pathophysiological conditions, altered shear stress, oxidative stress, and inflammatory factors disrupt Ca^2+^ homeostasis, leading to endothelial dysfunction [177]. This impairment affects vascular tone, increases inflammation, and drives endothelial-to-myofibroblast transition, consequently contributing to myocardial ischemia and cardiac hypertrophy [177]. Given the importance of Ca^2+^ signaling in vascular function, this review summarized Ca^2+^-related ion channels in ECs and explores how SGLT2i alleviate HF-induced cardiac dysfunction by modulating these endothelial ion channels.

Although most preclinical studies focus on the direct effect of SGLT2 on CMs, increasing experimental evidence highlights the significant contribution of endothelial ion channels to SGLT2i-mediated cardioprotection. Our review suggests that SGLT2i-induced modulation of endothelial ion channel function directly alleviates endothelial damage and improves intracellular interaction between ECs and CMs. This is evidenced by improved eNOS/NO balance and cardiomyocyte relaxation, reduction in intracellular Ca^2+^, ROS generation, and inflammation. These endothelial protective effects of SGLT2i are likely independent of SGLT2, indicating that SGLT2i directly target endothelial ion channels such as NHE1 and NCX.

The expression of SGLT2 in cardiac ECs and the extent to which SGLT2i exert their beneficial effects through SGLT2 inhibition in the heart are debatable. Some studies have reported that human ECs from healthy hearts, HF hearts and large vessels do not express SGLT2 [103, 114, 158]. In contrast, other studies have detected a low level of SGLT2 expression in human cardiac ECs [101, 142]. Low-grade inflammation or high-glucose levels have been shown to increase SGLT2 expression in both human and porcine ECs, and suggested a positive link between SGLT2 and endothelial inflammation [36, 115, 126].

Several initial studies conducted by our group identified the off-target effect of SGLT2i in the heart [8, 157]. Nevertheless, these studies did not demonstrate that these effects were independent of SGLT2, as SGLT2 was not examined. Treatment with three different SGLT2i (EMPA, DAPA, and ertugliflozin (ERTU)) strongly increased urinary glucose levels to a similar extent, indicating that all three inhibitors were equally effective in inhibiting SGLT2 [121]. However, only EMPA and DAPA, but not ERTU, reduced infarct size following IRI, suggesting that their beneficial effects were unrelated to glucose excretion via SGLT2 inhibition in the kidney [121]. Since SGLT2 was not tested, they also could not directly demonstrate that the protective effects of SGLT2i were independent of SGLT2.

Our group generated global SGLT2 KO mice, providing the first direct proof that the cardioprotective effects of EMPA persist in the absence of SGLT2 expression [27]. Similarly, the cardioprotective effects of DAPA also remained in SGLT2 KO mice [172], directly indicating that the cardioprotective effects of SGLT2i are mediated by mechanisms other than SGLT2 inhibition. Our findings suggest that SGLT2i may directly suppress NHE1/Na^+^/NCX/Ca^2+^ in cardiac ECs, thereby alleviating HF-induced cardiac dysfunction [22, 81, 94].

Baker et al. failed to find the inhibition of NHE1 by SGLT2i in hearts and suggested that the protective effect of SGLT2i was mediated by increasing preload [11, 12]. In this study, healthy pigs treated with CANA (oral, 300 mg, single day) showed an increase in end-diastolic volume (preload) during ischemia compared to the control group [11]. A similar effect was observed with acute intravenous CANA treatment (1 mg/kg, 15-30 min before ischemia), while treatment with cariporide (0.03 mg/kg) had a modest but statistically insignificant impact on end-diastolic volume [12]. An in vitro study showed that CANA, at concentrations ranging from 0.12–10 µM, did not inhibit NHE1 activity in AP-1 cells or human iPSC (Induced pluripotent stem cells)-derived CMs [12]. These findings argued against the possibility that SGLT2i exert cardioprotective effects via NHE1 inhibition [11, 12]. The varying effects of SGLT2i in hearts may be caused by different experimental conditions [25]. First, the in vivo study used a relatively low concentration of cariporide (~ 1 µM) but a significantly high concentration of CANA (~ 30 µM), which does not reflect clinically relevant dosing [39]. Moreover, in the in vitro study, 1.1 µM of cariporide was the lowest concentration required to inhibit NHE1, while 10 µM completely inhibited the NHE1 activity [12]. This might suggest that the cariporide exposure in vivo was insufficient to reveal the cardioprotective effects of NHE1 inhibition. Secondly, the authors studied the inhibitory effect of CANA on NHE1 in iPSC derived CMs, whereas other studies used freshly isolated CMs [8, 157]. The in vitro expansion and differentiation of iPSC can induce genetic mutations which potentially affect the functionality of iPSC lines as well as downstream effects [112]. Notably, the beneficial effect of SGLT2i on contractility was only observed after prolonged human iPSC-derived CMs culturing, highlighting that alterations in culturing conditions may abolish the effects of SGLT2i [170]. Furthermore, the studies by Baker et al. [11, 12] did not investigate the endothelial protective effect of CANA and, therefore, could not prove whether CANA improves cardiac function by inhibiting endothelial NHE1.

SGLT2i may affect ion homeostasis by regulating AMPK, a key regulator of glucose metabolism [43]. The function of AMPK linked to cardiovascular diseases has been reviewed elsewhere [135, 174]. Activation of AMPK can protect heart cells (ECs, CMs, and fibroblasts) from damage by increasing glucose uptake and regulating Ca^2+^ channels [135]. SGLT2i can reduce endothelial inflammation and oxidative stress, and improve eNOS activity as well as endothelium-dependent vasodilation in an AMPK-dependent manner [100, 159, 186]. In CMEC isolated from diabetic mice, EMPA reduced mitochondria ROS overproduction, adhesion molecules expression and improved eNOS phosphorylation through AMPK activation via an increased AMP/ATP ratio which triggered AMPK activation [186]. Cui et al. revealed the link between AMPK and Ca^2+^ homeostasis in lipopolysaccharide (LPS)-exposed ECs, showing that AMPK activation improved ATP content and SERCA2a (an ATP-driven Ca^2+^ reuptake channel in the ER. Increased SERCA2a reduced LPS-mediated Ca^2+^ overload, mitochondrial ROS production and mitochondrial apoptosis [34]. SGLT2i have been shown to regulate ion channels through AMPK activation in CMs as well as fibroblasts. DAPA-mediated AMPK activation contributed to the reduction of NHE activity in fibroblasts [179]. There is a positive link between upregulated Hsp70 (heat shock protein-70)/NHE1 association and NHE1 activity [72]. In LPS-exposed cardiofibroblasts, DAPA reduced NHE1 mRNA level and inhibited Hsp70 /NHE1 association by activating AMPK, suggesting that DAPA might affect post-translation of NHE1 through activating AMPK [179]. In addition, EMPA reduced intracellular pH, and increased AMPK activation as well as ATP production in CMs, similar to the effects of the NHE1 inhibitor cariporide [46]. These findings indicate a potential causal relationship between SGLT2i-mediated AMPK activation and NHE1 activity, highlighting the possibility that SGLT2i may reduce NHE1 activity by the activation of AMPK. Therefore, these studies exploring SGLT2i-mediated AMPK activation and NHE1 regulation in other cardiac cell types could provide valuable insights into the potential effects of SGLT2i on ECs.

## Conclusion

The pathophysiology of ED plays a crucial role in the progression of HF. In HF, the imbalance between NO bioavailability and oxidative stress in ECs contributes to impaired coronary circulation, reduced ventricular function, and increased vascular stiffness. Endothelial ion homeostasis is closely linked to ED, and SGLT2i have been reported to exert cardioprotective effects in HF, potentially through modulating endothelial ion channels. Given the limited understanding of endothelial ion channels and their role in HF, further in vitro and in vivo studies are needed to fully elucidate the potential effects of SGLT2i on endothelial ion channels. These studies would be crucial for understanding how SGLT2 inhibitors improve cardiac function at the endothelial level.

## Data Availability

No data was used for the research described in the article.

## Abbreviations

AMPK: AMP-activated protein kinase
Ca^2+^: Calcium
CaMKII: Calcium/calmodulin-dependent protein kinase II
Cardiomyocytes: CMs
CMEC: Coronary microvascular endothelial cells
K_Ca_: Calcium-activated potassium channels
CV: Cardiovascular
ECs: Endothelial cells
ED: Endothelial dysfunction
EDH: Endothelium-dependent hyperpolarization
EDHF: Endothelium-dependent hyperpolarization factors
HF: Heart failure
HFpEF: Heart failure with mildly preserved ejection fraction
HFmEF: Heart failure with mildly reduced ejection fraction
HFrEF: Heart failure with reduced ejection fraction
HCA: Human coronary arterioles
HCAECs: Human coronary artery endothelial cells
HUVECs: Human umbilical vein endothelial cells
IK_Ca_: Intermediate conductance calcium-activated potassium channels
[Ca^2+^]_i_: Intracellular calcium concentration
[Na^+^]_i_: Intracellular sodium concentration
BK_Ca_: Large conductance calcium-activated potassium channels
LVEF: Left ventricular ejection fraction
NO: Nitric oxide
NOS: NO synthase
K^+^: Potassium
ROS: Reactive oxygen species
RAAS: Renin–angiotensin–aldosterone system
NCXrm: Reverse mode of NCX
SK_Ca_: Small conductance calcium-activated potassium channels
Na^+^: Sodium
NCX: Sodium-calcium exchanger
SGLT2i: Sodium-glucose cotransporter 2 inhibitors
NHE: Sodium-hydrogen exchanger
SNS: Sympathetic nervous system
TRP: Transient receptor potential
TRPV: Transient receptor potential vanilloid
TAC: Transverse aortic constriction
T2DM: Type 2 diabetes mellitus
VSMC: Vascular smooth muscle cells
